# Association of insomnia symptoms and trajectories with the risk of functional disability: a prospective cohort study

**DOI:** 10.1186/s12877-024-05108-9

**Published:** 2024-06-05

**Authors:** Qing-Mei Huang, Jia-Hao Xie, Huan Chen, Hao-Yu Yan, Jian Gao, Zhi-Hao Li, Xiang Gao, Virginia Byers Kraus, Chen Mao

**Affiliations:** 1 Department of Epidemiology, School of Public Health, Southern Medical University, Guangzhou, 510515 Guangdong China; 2 Department of Nutritional Sciences, The Pennsylvania State University, University Park, PA USA; 3 Duke Molecular Physiology Institute, Division of Rheumatology, Department of Medicine, Duke University School of Medicine, Durham, NC USA

**Keywords:** Insomnia symptoms, Trajectory, Functional disability, Cohort study

## Abstract

**Background:**

There is limited understanding regarding prospective associations of insomnia symptoms and trajectories with functional disability. We aimed to investigate the associations of insomnia symptoms and trajectories with functional disability.

**Method:**

A total of 13 197 participants were eligible from the Health and Retirement Study. Insomnia symptoms included non-restorative sleep, difficulty initiating sleep, early morning awakening, and difficulty maintaining sleep. We also identified four distinct trajectories of insomnia symptoms: low, decreasing, increasing, and high insomnia symptoms. Functional status was assessed through activities of daily living (ADL) and instrumental activities of daily living (IADL).

**Results:**

Participants experiencing one (HR, 1.21; 95% CI, 1.13–1.29), two (HR, 1.43; 95% CI, 1.29–1.57), or three to four (HR, 1.41; 95% CI, 1.25–1.60) insomnia symptoms had a higher risk of ADL disability than asymptomatic respondents. Similarly, participants with one or more insomnia symptoms had a higher risk of IADL disability. Furthermore, using the trajectory with low insomnia symptoms as the reference, decreasing insomnia symptoms (HR, 1.22; 95% CI, 1.12–1.34), increasing insomnia symptoms (HR, 1.21; 95% CI, 1.05–1.41), and high insomnia symptoms (HR, 1.36; 95% CI, 1.18–1.56) were all associated with an increased risk of ADL disability.

**Conclusion:**

Both a single measurement and dynamic trajectory of insomnia symptoms are associated with the onset of ADL disability. Increased awareness and management of insomnia symptoms may contribute to the prevention of functional disability occurrence.

**Supplementary Information:**

The online version contains supplementary material available at 10.1186/s12877-024-05108-9.

## Introduction

Functional disability is a significant public health concern among older adults, usually assessed by activities of daily living (ADL) and instrumental activities of daily living (IADL). In 2019, over 60% of older adults in the United States reported difficulties in performing at least one daily function [1, 2]. Older adults experiencing functional disabilities often utilize services extensively, resulting in elevated healthcare expenditures [3, 4]. Moreover, the presence of functional disability exhibits a substantial correlation with subsequent occurrences of falls, injury, dependency, cognitive decline, and death [5, 6]. Millions of healthy years of life are lost as a result of functional disability for Americans aged 50 or older [7]. Thus, identifying modifiable risk factors, can be crucial to reducing the incidence of functional disability and improving the quality of later life.

Insomnia, a clinically heterogeneous disorder, is frequently diagnosed through the subjective assessment of sleep quality [8, 9]. Insomnia symptoms, such as non-restorative sleep, difficulty initiating sleep, early morning awakening, and difficulty maintaining sleep, are prevalent among older adults, with up to 75% of older adults experiencing at least one insomnia symptom annually [10]. Insomnia is often associated with a range of physical and psychiatric comorbidities, including frailty, depression, and falls [11–13], which are acknowledged as well-established risk factors for functional disability [14–16]. Nonetheless, little is known about the effects of insomnia symptoms on future risk of functional disability. Moreover, the trajectory of insomnia symptoms over time exhibits considerable variability among individuals [17]. Some individuals may have persistent severe insomnia symptoms, while others may initially exhibit mild symptoms that gradually worsen. Alternatively, some individuals with severe insomnia symptoms experience subsequent symptom amelioration. These different insomnia symptom trajectories might differentially predict functional disability risk. Given the limited understanding of prospective associations between insomnia symptoms, particularly the trajectories of insomnia symptoms, and functional disability, it is imperative to conduct a study that covers a large community-dwelling population with a relatively extended follow-up period to better understand the association of insomnia symptoms and trajectories with functional disability.

Accordingly, we used longitudinal population-based cohort data from the Health and Retirement Study (HRS) to investigate the associations of insomnia symptoms and the trajectories of insomnia symptoms with functional disability.

## Methods

### Study design and participants

The study population and design of the HRS have been previously described [18]. Briefly, the HRS is a population-based cohort of community-dwelling adults in the United States, along with their spouses of any age. Extensive demographic and health information have been collected through biennial interviews starting from cohort entry and continuing until the occurrence of death or dropout. The HRS was approved by the Institutional Review Board at the University of Michigan and the National Institute on Aging (HUM00061128). Verbal informed consent is obtained from all participants in the HRS prior to their involvement in the study.

In the current study, participants were from one wave of the HRS (2002) and were limited to those aged 50 years or older at baseline (*n* = 17 758). In the insomnia symptoms association analysis, participants with missing data on insomnia symptoms at baseline (2002), those reporting ADL or IADL disability at baseline, or those lost to follow-up at any time point were excluded, leaving 13 197 participants who were included. In the trajectories of insomnia symptoms association analysis, participants with missing data on insomnia symptoms at the three examination rounds in 2002 (baseline), 2004 (visit 1), and 2006 (visit 2), those reporting ADL or IADL disability at the three examination rounds, or those lost to follow-up at any time point were excluded, leaving 8 823 participants who were included (eFigure 1).

### Assessment of insomnia symptoms

Participants were asked about insomnia symptoms at each wave. Questions about insomnia symptoms assessed difficulties in initiating and maintaining sleep, early morning awakening, and non-restorative sleep [19–21]. Participants were asked how often they have trouble with ‘falling asleep’, ‘waking up during the night’, and ‘waking up too early and not being able to fall asleep again’, and how often they feel ‘really rested’ when they wake up in the morning. The response options for each question included ‘most of the time’, ‘sometimes’, and ‘rarely or never’. We defined participants as experiencing insomnia symptoms when they answered ‘most of the time’ or ‘sometimes’ to the first three questions, and answered ‘rarely or never’ or ‘sometimes’ to the last question, as described by prior studies [19, 22]. Each insomnia symptom status was then represented with a binary variable, ‘yes vs. no’, in the analyses. These items that participants were asked about their insomnia symptoms have been widely used in the literature [19, 22] and were strongly correlated with several established multiple-item scales [23]. To examine the quantity of insomnia symptoms, we categorized participants based on the number of reported symptoms. This was performed by summing across the four symptoms and categorizing the respondents into four groups: 1) experiencing no symptoms, 2) experiencing one symptom, 3) experiencing two symptoms, and 4) experiencing three to four symptoms. To examine the trajectories of insomnia symptoms from 2002 to 2006, responses ranged from 2 (‘most of the time’) to 0 (‘rarely or never’) for the first three questions and ranged from 0 (‘most of the time’) to 2 (‘rarely or never’) for the last question. We summed values from the four items to compute an insomnia score for each wave, ranging from 0 to 8 [24].

### Assessment of functional disability

Functional status was measured by ADL and IADL [25, 26]. Participants in HRS were asked if they required assistance with any of the six ADLs (dressing, bathing, eating, using the toilet, getting in/out of bed, and walking across a room) or the five IADLs (using the phone, managing money, taking medications, shopping for groceries, and preparing hot meals). In accordance with previous studies [27], ADL and IADL were dichotomized into no limitation or at least one limitation. Once one or more limitations emerged at follow-up, those participants would be defined as ‘incident ADL/IADL disability’.

### Covariates

Three variable sets were considered potential confounding factors and were defined as follows. The first set defined demographic variables: age, sex (male or female), ethnicity (non-Hispanic white, non-Hispanic black, Hispanic, or other), education level (less than college or college and above), and body mass index (BMI). The second set included lifestyle factors: current smoking (yes or no), alcohol drinking (yes or no), and regular exercise (yes or no). The third set included self-reported physician-diagnosed diseases: hypertension (yes or no), diabetes (yes or no), stroke (yes or no), cancer (yes or no), chronic lung disease (yes or no), heart disease (yes or no), and depressive symptom (yes or no). BMI was equal to weight divided by squared body height in kilograms /m^2^. Depressive symptoms were assessed using an 8-item version of the Center for Epidemiologic Studies Depression Scale, with scores of ≥ 4 regarded as indicating depressive symptoms.

### Statistical analysis

The baseline characteristics of the included participants were summarized by the number of insomnia symptoms as numbers (percentages) for categorical variables and as means (SDs) for normally distributed continuous variables. Differences in characteristics between the number of insomnia symptoms or the trajectories of insomnia symptoms were tested using analysis of variance or χ^2^ tests. The multiple imputation method was used to correct the missing values and reduce the possibility of inferential bias.

We first used latent class trajectory models to identify trajectories of insomnia symptoms over time. This is a specialized form of finite mixture modeling and is designed to identify latent classes of individuals following similar progressions of a determinant over time [28]. Our models used second-order polynomials. For every participant, we calculated the posterior probabilities for each trajectory, and we assigned participants post hoc to the trajectory with the highest probability. We estimated the best-fitting number of trajectories based on a minimum Bayesian Information Criterion [29], while maintaining the posterior probabilities by class (> 0.70) and class size (≥ 2% of the population). To facilitate interpretability, we assigned labels to the trajectories on the basis of their modeled graphic patterns.

To assess the risk of functional disability, the survival model time zero was the examination date of the first examination round (baseline) in the insomnia symptoms association analysis and the examination date of the third examination round in the trajectories of insomnia symptoms association analysis. Cox proportional hazards regression models were used to assess the associations of insomnia symptoms (the number and type of insomnia symptoms) and the trajectories of insomnia symptoms with the risk of incident ADL or IADL disability. The hazard ratios (HRs) along with the 95% confidence intervals (CIs) were calculated after multiple comparisons using Bonferroni correction. Proportional hazards assumptions were not violated when assessed using Schoenfeld residuals (*P* > 0.05). For all analyses, we fitted two models: model 1 was unadjusted. Model 2 was adjusted for age, sex, ethnicity, education level, BMI, current smoking, alcohol drinking, regular exercise, hypertension, diabetes, stroke, cancer, chronic lung disease, heart disease, and depressive symptom.

Additionally, we conducted subgroup analyses stratified by sex (male and female). Sensitivity analyses were performed to exclude participants with missing covariate data and to exclude those who developed ADL or IADL disability during the first two years of follow-up to account for the possibility of reverse causation. We further explored the associations of insomnia symptoms and trajectories with each activity explored by ADL and IADL.

All statistical analyses were performed in R software, version 4.3.2 (R Project for Statistical Computing). Reported *P* values were two-tailed, and *P* < 0.05 was considered statistically significant.

## Results

### Baseline characteristics

Table 1 shows the baseline characteristics of the study participants according to the number of insomnia symptoms. Of the 13 197 eligible HRS sample members with a median age of 67.4 years, 56.8% were female. Of the 13 197 participants, the proportion of those with at least one insomnia symptom was 33.4%.

**Table 1:** Baseline characteristics of study participants according to number of insomnia symptoms

Characteristic	Total (*N* = 13 197)	Number of Insomnia Symptoms				*P* Value
		0 (*n* = 8 788)	1 (*n* = 2 919)	2 (*n* = 936)	3–4 (*n* = 554)	
Age, mean (SD), y	67.4 (9.0)	67.1 (8.9)	68.0 (9.0)	67.7 (9.1)	67.0 (9.0)	< 0.001
Female	7 491 (56.8)	4 877 (55.5)	1 643 (56.3)	592 (63.2)	379 (68.4)	< 0.001
Ethnicity						< 0.001
Non-Hispanic white	10 324 (78.2)	6 708 (76.3)	2 392 (81.9)	768 (82.1)	456 (82.3)	
Non-Hispanic black	1 622 (12.3)	1 173 (13.3)	311 (10.7)	85 (9.1)	53 (9.6)	
Hispanic	999 (7.6)	729 (8.3)	176 (6.0)	60 (6.4)	34 (6.1)	
Other	252 (1.9)	178 (2.0)	40 (1.4)	23 (2.5)	11 (2.0)	
Education level						< 0.001
Less than college	3 438 (22.3)	585 (19.1)	1 178 (24.2)	206 (31.0)	182 (23.2)	
College and above	2 704 (20.5)	1 880 (21.4)	572 (19.6)	175 (18.7)	77 (13.9)	
BMI, mean (SD), kg/m^2^	27.1 (5.0)	27.0 (4.9)	27.1 (5.0)	27.1 (5.2)	27.4 (5.3)	0.307
Current smoking	1 814 (13.7)	1 239 (14.1)	362 (12.4)	136 (14.5)	77 (13.9)	0.118
Alcohol drinking	6 594 (50.0)	4 454 (50.7)	1 467 (50.3)	436 (46.6)	237 (42.8)	0.001
Regular exercise	6 191 (46.9)	4 299 (48.9)	1 321 (45.3)	388 (41.5)	183 (33.0)	< 0.001
Comorbidities						
Hypertension	6 245 (47.3)	4 093 (46.6)	1 429 (49.0)	451 (48.2)	272 (49.1)	0.108
Diabetes	1 853 (14.0)	1 186 (13.5)	448 (15.3)	137 (14.6)	82 (14.8)	0.077
Stroke	654 (5.0)	396 (4.5)	159 (5.4)	59 (6.3)	40 (7.2)	0.002
Cancer	1 571 (11.9)	977 (11.1)	387 (13.3)	135 (14.4)	72 (13.0)	0.001
Chronic lung disease	773 (5.9)	415 (4.7)	203 (7.0)	99 (10.6)	56 (10.1)	< 0.001
Heart disease	2 480 (18.8)	1 498 (17.0)	616 (21.1)	223 (23.8)	143 (25.8)	< 0.001
Depressive symptom	1372 (10.4)	590 ( 6.7)	370 (12.7)	217 (23.2)	195 (35.2)	< 0.001

Abbreviations: SD, standard deviation; BMI, body mass index. Values are numbers (percentages) unless stated otherwise

Compared with participants with no insomnia symptoms, those with at least one insomnia symptom were more likely to be female, non-Hispanic white, and less educated, and more likely to have a higher prevalence of stroke, cancer, chronic lung disease, and heart diseases; they were less likely to be heavy drinkers or regular exercisers. Participants with one or two insomnia symptoms tended to be older, while those with three or four insomnia symptoms tended to be younger. The baseline characteristics of the study participants according to the trajectories of insomnia symptoms are shown in eTable 1.

### Insomnia symptoms and functional disability

We observed 5 184 events of ADL disability and 4 712 events of IADL disability during a mean follow-up of 10 years. Table 2 shows the association between the cumulative number and type of insomnia symptoms and incident ADL disability. After adjusting for multiple confounding variables, participants experiencing one (HR, 1.21; 95% CI, 1.13–1.29), two (HR, 1.43; 95% CI, 1.29–1.57), or three to four (HR, 1.41; 95% CI, 1.25–1.60) insomnia symptoms had a higher risk of incident ADL disability compared to those not experiencing any insomnia symptoms. For each symptom individually, experiencing non-restorative sleep (HR, 1.31; 95% CI, 1.20–1.43), difficulty initiating sleep (HR, 1.30; 95% CI, 1.19–1.41), early morning awakening (HR, 1.20; 95% CI, 1.10–1. 31), or difficulty maintaining sleep (HR, 1.23; 95% CI, 1.16–1.32), was associated with a higher risk of incident ADL disability compared to those not experiencing the symptom.

**Table 2:** Association between the cumulative number and type of insomnia symptoms and ADL disability

Respondent Characteristics	Events/Total	Model 1^a^		Model 2^b^	
		HR (95% CI)	*P* Value	HR (95% CI)	*P* Value
Number of insomnia symptoms (ref: no symptoms)					
1	1 271/2 919	1.30 (1.22–1.39)	< 0.001	1.21 (1.13–1.29)	< 0.001
2	475/936	1.66 (1.50–1.82)	< 0.001	1.43 (1.29–1.57)	< 0.001
3–4	275/554	1.65 (1.46–1.87)	< 0.001	1.41 (1.25–1.60)	< 0.001
Individual insomnia symptoms					
Non-restorative sleep (ref: no)	637/1 356	1.37 (1.26–1.49)	< 0.001	1.31 (1.20–1.43)	< 0.001
Difficulty initiating sleep (ref: no)	604/1 182	1.55 (1.43–1.69)	< 0.001	1.30 (1.19–1.41)	< 0.001
Early morning awakening (ref: no)	590/1 253	1.38 (1.27–1.51)	< 0.001	1.20 (1.10–1.31)	< 0.001
Difficulty maintaining sleep (ref: no)	1 297/2 807	1.34 (1.26–1.43)	< 0.001	1.23 (1.16–1.32)	< 0.001

Abbreviations: HR, hazard ratio; CI, confidence interval

^a^ Unadjusted

^b^ Adjusted for age, sex, ethnicity, education level, current smoking, alcohol drinking, BMI, regular exercise, hypertension, diabetes, stroke, cancer, chronic lung disease, heart disease, and depressive symptom

We observed similar patterns of results for IADL disability (Table 3). After adjusting for multiple confounding variables, participants experiencing one (HR, 1.14; 95% CI, 1.07–1.23), two (HR, 1.21; 95% CI, 1.09–1.35), or three to four (HR, 1.18; 95% CI, 1.03–1.35) insomnia symptoms had a higher risk of incident IADL disability compared to those not experiencing any insomnia symptoms. For each symptom individually, experiencing non-restorative sleep (HR, 1.16; 95% CI, 1.05–1.27), early morning awakening (HR, 1.12; 95% CI, 1.03–1.23), or difficulty maintaining sleep (HR, 1.15; 95% CI, 1.07–1.23) was associated with a higher risk of incident IADL disability compared with those not experiencing the symptom.

**Table 3:** Association between the cumulative number and type of insomnia symptoms and IADL disability

Respondent Characteristics	Events/Total	Model 1^a^		Model 2^b^	
		HR (95% CI)	*P* Value	HR (95% CI)	*P* Value
Number of insomnia symptoms (ref: no symptoms)					
1	1 150/2 919	1.25 (1.16–1.34)	< 0.001	1.14 (1.07–1.23)	< 0.001
2	402/936	1.42 (1.28–1.58)	< 0.001	1.21 (1.09–1.35)	< 0.001
3–4	231/554	1.42 (1.24–1.62)	< 0.001	1.18 (1.03–1.35)	< 0.001
Individual insomnia symptoms					
Non-restorative sleep (ref: no)	537/1 356	1.21 (1.10–1.32)	< 0.001	1.16 (1.05–1.27)	0.002
Difficulty initiating sleep (ref: no)	497/1 182	1.33 (1.21–1.46)	< 0.001	1.06 (0.96–1.16)	0.246
Early morning awakening (ref: no)	525/1 253	1.32 (1.20–1.44)	< 0.001	1.12 (1.03–1.23)	0.012
Difficulty maintaining sleep (ref: no)	1 146/2 807	1.25 (1.17–1.34)	< 0.001	1.15 (1.07–1.23)	< 0.001

Abbreviations: HR, hazard ratio; CI, confidence interval

^a^ Unadjusted

^b^ Adjusted for age, sex, ethnicity, education level, current smoking, alcohol drinking, BMI, regular exercise, hypertension, diabetes, stroke, cancer, chronic lung disease, heart disease, and depressive symptom

### Trajectories of insomnia symptoms and functional disability

We identified four distinct trajectories of insomnia symptoms in the 8 823 individuals with data related to baseline and two follow-up visits (eFigure 2) as follows (*n*, [%]): maintained a low insomnia symptom score throughout the follow-up (‘low insomnia symptoms’; 5 320, [60.3%]); had a moderately high starting score but then remitted (‘decreasing insomnia symptoms’; 2 279, [25.8%]); had a low starting score that steadily increased throughout the follow-up (‘increasing insomnia symptoms’; 565, [6.4%]); and maintained a high score throughout (‘high insomnia symptoms’; 659, [7.5%]). Tables 4 and 5 show the association of the trajectories of insomnia symptoms with ADL disability and IADL disability, respectively. Using the trajectory with low insomnia symptoms as the reference, decreasing insomnia symptoms (HR, 1.22; 95% CI, 1.12–1.34), increasing insomnia symptoms (HR, 1.21; 95% CI, 1.05–1.41), and high insomnia symptoms (HR, 1.36; 95% CI, 1.18–1.56) were all associated with an increased risk of ADL disability. Only the trajectory with decreasing insomnia symptoms was associated with a higher risk of incident IADL disability (HR, 1.14; 95% CI, 1.04–1.25).

**Table 4:** Association Between the Trajectories of Insomnia Symptoms and ADL Disability

Trajectories of Insomnia Symptoms	Events/Total	Model 1^a^		Model 2^b^	
		HR (95% CI)	*P* Value	HR (95% CI)	*P* Value
Low	1 437/5 320	1.00 (reference)	-	1.00 (reference)	-
Decreasing	810/2 279	1.36 (1.25–1.49)	< 0.001	1.22 (1.12–1.34)	< 0.001
Increasing	202/565	1.42 (1.22–1.64)	< 0.001	1.21 (1.05–1.41)	0.011
High	253/659	1.55 (1.36–1.77)	< 0.001	1.36 (1.18–1.56)	< 0.001

Abbreviations: HR, hazard ratio; CI, confidence interval

^a^ Unadjusted

^b^ Adjusted for age, sex, ethnicity, education level, current smoking, alcohol drinking, BMI, regular exercise, hypertension, diabetes, stroke, cancer, chronic lung disease, heart disease, and depressive symptom

**Table 5:** Association Between the Trajectories of Insomnia Symptoms and IADL Disability

Trajectories of Insomnia Symptoms	Events/Total	Model 1^a^		Model 2^b^	
		HR (95% CI)	*P* Value	HR (95% CI)	*P* Value
Low	1 374/5 320	1.00 (reference)	-	1.00 (reference)	-
Decreasing	722/2 279	1.26 (1.15–1.37)	< 0.001	1.14 (1.04–1.25)	0.007
Increasing	183/565	1.30 (1.11–1.52)	0.001	1.08 (0.92–1.26)	0.354
High	202/659	1.20 (1.03–1.39)	0.017	1.04 (0.89–1.21)	0.660

Abbreviations: HR, hazard ratio; CI, confidence interval

^a^ Unadjusted

^b^ Adjusted for age, sex, ethnicity, education level, current smoking, alcohol drinking, BMI, regular exercise, hypertension, diabetes, stroke, cancer, chronic lung disease, heart disease, and depressive symptom

### Sensitivity analyses

The association of insomnia symptoms and trajectories with ADL and IADL disability were generally similar across sex (eTables 2–5). The association of insomnia symptoms and trajectories with ADL and IADL disability was robust and stable in all sensitivity analyses. Similar findings were observed when excluding participants with missing covariates data (eTables 6–9) or excluding participants who developed ADL or IADL disability during the first two years of follow-up (eTables 10–13). The associations of insomnia symptoms and and trajectories with each activity explored by ADL and IADL were showed in eTables 14–17.

## Discussion

In this large, nationally representative prospective study of adults in the United States, we found that participants who experienced more insomnia symptoms had a higher risk of functional disability. Furthermore, we identified four distinct trajectories of insomnia symptoms, characterized by low, decreasing, increasing, and high insomnia symptoms trajectories. The trajectories characterized by decreasing, increasing, and high insomnia symptoms were associated with a higher risk of ADL disability, while the trajectory characterized by decreasing insomnia symptoms was associated with a higher risk of IADL disability.

Some previous cross-sectional and longitudinal studies that had limited sample sizes and used only a few insomnia symptom assessments showed some positive associations between insomnia symptoms and functional disability [22, 30–32]. A cross-sectional study of over 9 000 older adults found that elevated self-reported measures of poor nighttime sleep and related daytime complaints were associated with self-reported impairment in ADLs and related tasks [30]. Cross-sectional findings from the Cardiovascular Health Study indicated that complaints of frequent awakenings and daytime sleepiness were independently associated with self-reported IADL impairment among older women [31]. In addition, a longitudinal study among 908 non-disabled older adults found that waking up at night and not feeling rested in the morning were associated with incident ADL disability [22]. Similarly, another longitudinal study among 817 women showed that women in the highest quartile of awakening after sleep onset had 74% higher odds of incident IADL disability than those in the lowest quartile [32]. Furthermore, our study found that insomnia symptoms affect ADL more than IADL, given that ADLs are routine activities that people do every day without needing assistance, while IADLs relate to independent living in the community, which often require more complex social interactions. Taken together, these findings suggest that insomnia symptoms potentially contribute to functional disability development.

To the best of our knowledge, no study has provided a clear understanding of the trajectory of insomnia symptoms over time and its dynamic relationship to the onset of functional disability. It is generally accepted that sleep quality worsens in old age [8, 33, 34], but there is heterogeneity in changes in sleep problems over time. Using a latent class trajectory model, four distinct insomnia symptom trajectories were identified in our study based on changes in participants’ insomnia symptoms using 3-time points over 4 years. We noted that participants with decreasing, increasing, and high insomnia symptoms had a significantly higher incidence of ADL disability, while individuals with decreasing insomnia symptoms had a higher risk of IADL disability. A study among 1 627 postmenopausal women reported that those with a consistently high likelihood of insomnia symptoms, and those with a decreased likelihood of insomnia symptoms, had slower gait speed than those with a consistently low likelihood of insomnia symptoms [35]. However, in this study, physical function was assessed using a 40-foot walk and a 4-meter walk, which cannot comprehensively measure the ability of older adults to perform daily activities. We extend these findings by demonstrating these associations in a large sample of older adults using systematic measures of ADL or IADL, and taking into account longitudinal information on the trajectories of insomnia symptoms in older adults.

The precise mechanisms by which insomnia symptoms are correlated with the onset of functional disability remain largely unidentified. Insomnia symptoms provoke a state of heightened arousal throughout both sleep and wakefulness, often co-occurring with increased heart rate, diminished heart rate variability, heightened blood pressure, and increased cortisol secretion. These symptoms may culminate in the onset of deleterious health conditions, encompassing insulin resistance, diabetes, hypertension, coronary heart disease, as well as mental health disorders, including anxiety and depression [36–38]. The direct and indirect detrimental health effects of insomnia may mediate or moderate the relationships between insomnia symptoms and functional disability. For instance, a growing body of evidence indicates that individuals with conditions such as hypertension, myocardial infarction, and diabetes are significantly more likely to develop functional disability [39–41]. Additionally, the association between insomnia symptoms and ADL or IADL disability may be linked to musculoskeletal system impairment. Research indicates that sleep deprivation can induce inflammation and impaired energy metabolism, resulting in musculoskeletal pain and tissue recovery impairment, as well as fibromyalgia (a widespread musculoskeletal pain disorder). Consequently, this can culminate in functional disability among older adults [42–44]. Moreover, there is evidence that inadequate sleep amplifies sensitivity to pain, leading to increased pain levels and reduced physical performance the subsequent morning [45].

Our study has several limitations. Firstly, insomnia symptoms were self-reported. While technologies such as actigraphy and polysomnography offer precise supplementary sleep data [46], measures derived from these devices are comparatively less sensitive and specific in recognizing insomnia symptoms [47]. Moreover, their application becomes impractical in extensive epidemiological investigations. Despite the potential for bias in subjective sleep perceptions, they meet the diagnostic criteria of insomnia disorder laid out in the DSM-5-TR [23]. Secondly, the survey assesses ADL and IADL as well as other health conditions, primarily relying on self-reported diagnoses from medical professionals. Nevertheless, despite the reliance on self-reporting, these measures have been validated for precise diagnoses of health conditions, particularly functional disabilities, and have consistently shown reliability across diverse populations in previous scientific studies [19, 48, 49]. Thirdly, this study only utilized data from the HRS dataset. Assessments of other secondary insomnia complaints and sleep quality such as total sleep time and sleep onset latency were absent in the dataset. Incorporating these variables could have significantly enhanced the depth and comprehensiveness of our analyses [36, 47, 50]. Fourthly, we do not have access to information about obstructive sleep apnoea syndrome (OSAS) which could be an essential contributing factor to insomnia symptoms such as non-restorative sleep, and the information about the use of psychotropic drugs, all of which might affect both sleep and physical function. Finally, the small sample size may have limited the statistical power, which might result in a non-significant association between high insomnia symptoms and IADL disability. Future research is warranted to increase the sample size to expand our findings.

## Conclusion

In this large community-based study, both a single measurement and dynamic trajectory of insomnia symptoms are associated with incident functional disability. The enhancement of public health awareness and the implementation of systematic screening for insomnia symptoms among the older adult population is crucial for mitigating the occurrence of functional disability.

### Electronic supplementary material

Below is the link to the electronic supplementary material.

Supplementary Material 1

## Data Availability

Data set: Available through the Health and Retirement Study (http://hrsonline.isr.umich.edu/).
