# Supplementary material for: Association of insomnia symptoms and trajectories with the risk of functional disability: a prospective cohort study
Source: BMC Geriatr. 2024 Jun 5;24:492. doi: 10.1186/s12877-024-05108-9 (PMC11151719; doi:10.1186/s12877-024-05108-9)
Supplement: Supplementary file 1 — Supplementary Material 1 [file 12877_2024_5108_MOESM1_ESM.docx]

**Supplementary Online Content**

**eFigure 1.** Inclusion and Exclusion Flowchart for the Study Participants.

**eFigure 2.** Trajectories of Insomnia Symptoms between 2002 to 2006.

**eTable 1.** Baseline Characteristics of Study Participants According to Trajectories of Insomnia Symptoms.

**eTable 2.** Association Between the Cumulative Number and Type of Insomnia Symptoms and ADL Disability Stratifying by Sex.

**eTable 3.** Association Between the Cumulative Number and Type of Insomnia Symptoms and IADL Disability Stratifying by Sex.

**eTable 4.** Association Between the Trajectories of Insomnia Symptoms and ADL Disability Stratifying by Sex.

**eTable 5.** Association Between the Trajectories of Insomnia Symptoms and IADL Disability Stratifying by Sex.

**eTable 6.** Association Between the Cumulative Number and Type of Insomnia Symptoms and ADL Disability after Excluding Participants Missing Covariate Data.

**eTable 7.** Association Between the Cumulative Number and Type of Insomnia Symptoms and IADL Disability after Excluding Participants Missing Covariate Data.

**eTable 8.** Association Between the Trajectories of Insomnia Symptoms and ADL Disability after Excluding Participants Missing Covariate Data.

**eTable 9.** Association Between the Trajectories of Insomnia Symptoms and IADL Disability after Excluding Participants Missing Covariate Data

**eTable 10.** Association Between the Cumulative Number and Type of Insomnia Symptoms and ADL Disability after Excluding Participants Who Developed ADL Disability During the First Two Years of Follow-up.

**eTable 11.** Association Between the Cumulative Number and Type of Insomnia Symptoms and IADL Disability after Excluding Participants Who Developed IADL Disability During the First Two Years of Follow-up.

.**eTable 12.** Association Between the Trajectories of Insomnia Symptoms and ADL Disability after Excluding Participants Who Developed ADL Disability During the First Two Years of Follow-up.

**eTable 13.** Association Between the Trajectories of Insomnia Symptoms and IADL Disability after Excluding Participants Who Developed IADL Disability During the First Two Years of Follow-up.

**eTable 14.** Association Between the Cumulative Number and Type of Insomnia Symptoms and each ADL disability.

**eTable 15.** Association Between the Cumulative Number and Type of Insomnia Symptoms and each IADL disability.

**eTable 16.** Association Between the Trajectories of Insomnia Symptoms and each ADL disability.

**eTable 17.** Association Between the Trajectories of Insomnia Symptoms and each IADL disability.


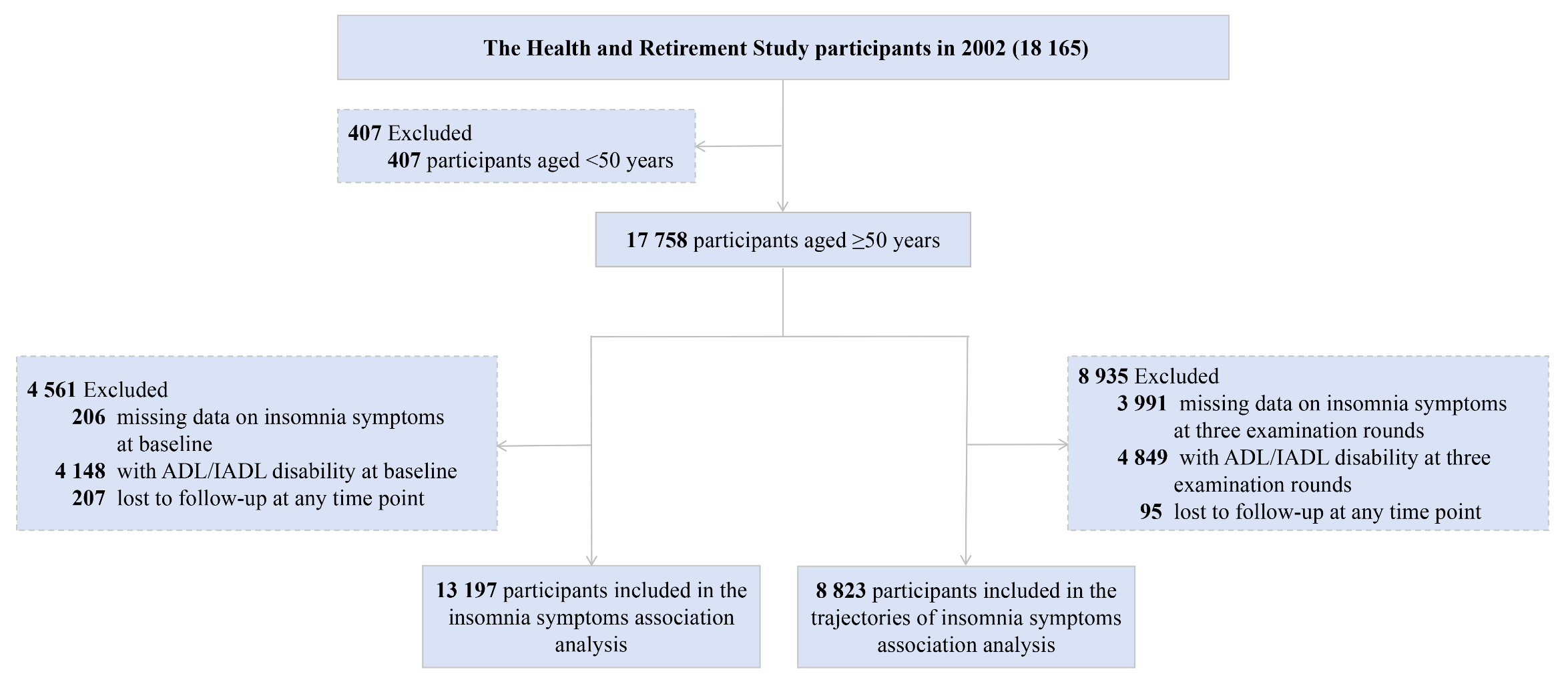


## eFigure 1: Flow Chart of Participants Enrolment.

Abbreviations: ADL, activities of daily living; IADL, instrumental activities of daily living.

##
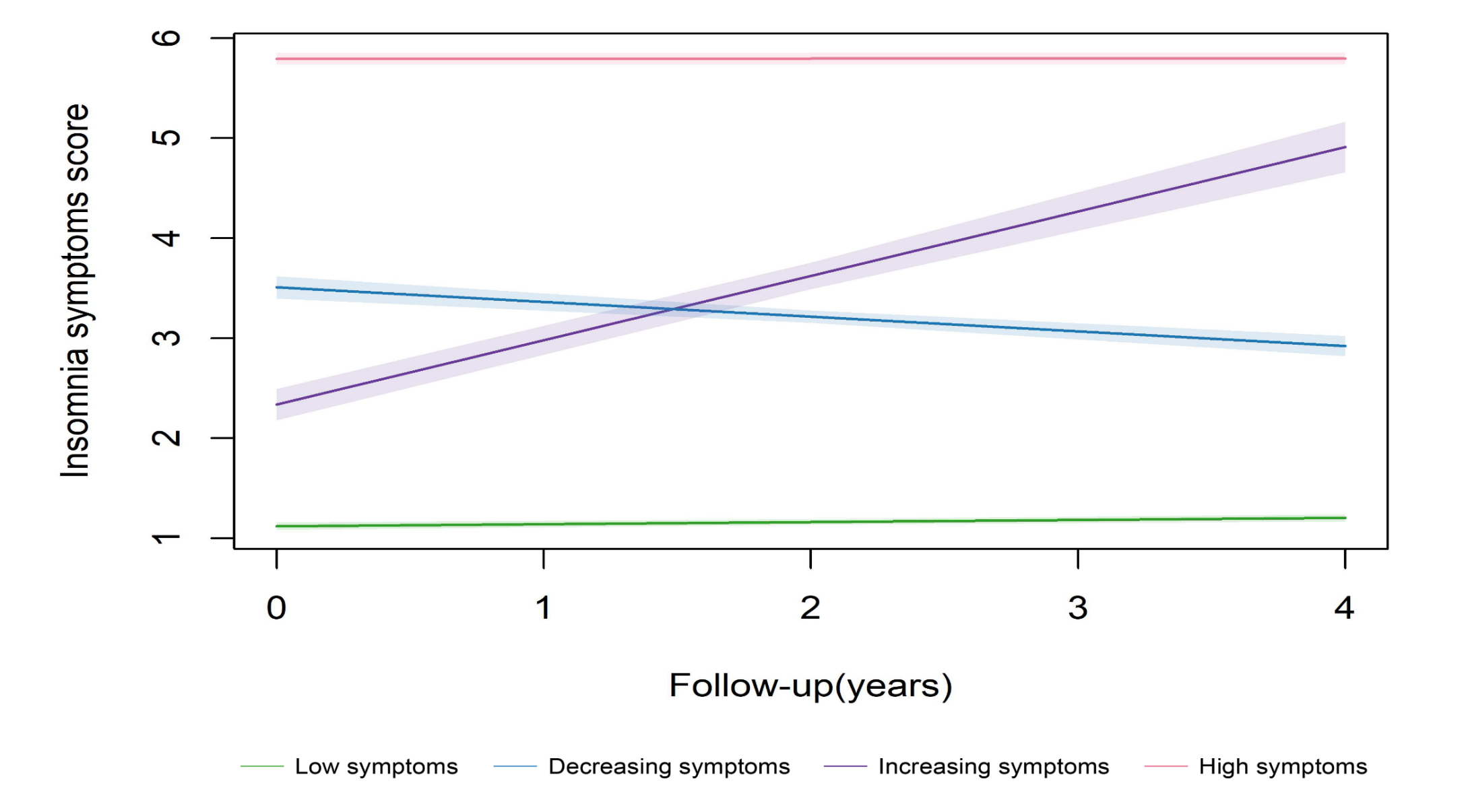


**eFigure 2:** **Trajectories of Insomnia Symptoms between 2002 to 2006.**

The figure shows trajectories of standardized insomnia symptoms scores over 4 years from 8 823 individuals, with four measures of insomnia symptoms. Shading around the lines represents confidence bands for the calculated trajectory.

## eTable 1. Baseline Characteristics of Study Participants According to Trajectories of Insomnia Symptoms.

| **Characteristic** | **Total**  **(N=8 823)** | **Trajectories of Insomnia Symptoms** | | | | ***P* Value** |
| --- | --- | --- | --- | --- | --- | --- |
|  |  | **Low**  **(n=5 320)** | **Decreasing**  **(n=2 279)** | **Increasing**  **(n=565)** | **High**  **(n=659)** |  |
| Age, mean (SD), y | 65.7 (8.0) | 65.6 (7.9) | 66.1 (8.1) | 66.0 (8.1) | 65.1 (8.1) | 0.015 |
| Female | 5 083 (57.6) | 2 755 (51.8) | 1 473 (64.6) | 377 (66.7) | 478 (72.5) | <0.001 |
| Ethnicity |  |  |  |  |  | <0.001 |
| Non-Hispanic white | 7 096 (80.4) | 4 208 (79.1) | 1 869 (82.0) | 454 (80.4) | 565 (85.7) |  |
| Non-Hispanic black | 941 (10.7) | 622 (11.7) | 221 (9.7) | 46 (8.1) | 52 (7.9) |  |
| Hispanic | 620 (7.0) | 392 (7.4) | 148 (6.5) | 50 (8.8) | 30 (4.6) |  |
| Other | 166 (1.9) | 98 (1.8) | 41 (1.8) | 15 (2.7) | 12 (1.8) |  |
| Education level |  |  |  |  |  | <0.001 |
| Less than college | 6 798 (77.0) | 3 978 (74.8) | 1 805 (79.2) | 465 (23.2) | 550 (83.5) |  |
| College and above | 2 025 (23.0) | 1 342 (25.2) | 474 (20.8) | 100 (82.3) | 109 (16.5) |  |
| BMI, mean (SD), kg/m^2^ | 27.1 (4.7) | 27.1 (4.7) | 27.0 (4.8) | 27.0 (4.6) | 27.1 (5.2) | 0.644 |
| Current smoking | 1 140 (12.9) | 696 (13.1) | 279 (12.2) | 73 (12.9) | 92 (14.0) | 0.639 |
| Alcohol drinking | 4 747 (53.8) | 2 917 (54.8) | 1 200 (52.7) | 307 (54.3) | 323 (49.0) | 0.022 |
| Regular exercise | 4 516 (51.2) | 2 837 (53.3) | 1 095 (48.0) | 300 (53.1) | 284 (43.1) | <0.001 |
| Comorbidities |  |  |  |  |  |  |
| Hypertension | 3 865 (43.8) | 2 260 (42.5) | 1 031 (45.2) | 270 (47.8) | 304 (46.1) | 0.013 |
| Diabetes | 994 (11.3) | 565 (10.6) | 287 (12.6) | 70 (12.4) | 72 (10.9) | 0.071 |
| Stroke | 306 (3.5) | 163 (3.1) | 90 (3.9) | 16 (2.8) | 37 (5.6) | 0.003 |
| Cancer | 936 (10.6) | 554 (10.4) | 233 (10.2) | 62 (11.0) | 87 (13.2) | 0.150 |
| Chronic lung disease | 380 (4.3) | 164 (3.1) | 144 (6.3) | 26 (4.6) | 46 (7.0) | <0.001 |
| Heart disease | 1 313 (14.9) | 722 (13.6) | 385 (16.9) | 74 (13.1) | 132 (20.0) | <0.001 |
| Depressive symptom | 700 ( 7.9) | 209 ( 3.9) | 272 (11.9) | 48 ( 8.5) | 171 (25.9) | <0.001 |

Abbreviations: SD, standard deviation; BMI, body mass index. Values are numbers (percentages) unless stated otherwise.

**eTable 2. Association Between the Cumulative Number and Type of Insomnia Symptoms and ADL Disability** **Stratifying by Sex.**

| **Respondent Characteristics** | **Female** | |  | **Male** | | ***P* for interaction** |
| --- | --- | --- | --- | --- | --- | --- |
|  | **HR (95% CI)** | ***P* Value** |  | **HR (95% CI)** | ***P* Value** |  |
| Number of insomnia symptoms (ref: no symptoms) | | | | | | 0.290 |
| 1 | 1.20 (1.10-1.30) | <0.001 |  | 1.24 (1.12-1.38) | <0.001 |  |
| 2 | 1.36 (1.20-1.54) | <0.001 |  | 1.57 (1.33-1.84) | <0.001 |  |
| 3-4 | 1.38 (1.19-1.61) | <0.001 |  | 1.50 (1.19-1.89) | 0.001 |  |
| Individual insomnia symptoms | | | | | |  |
| Non-restorative sleep (ref: no) | 1.31 (1.18-1.47) | <0.001 |  | 1.33 (1.16-1.52) | <0.001 | 0.995 |
| Difficulty initiating sleep (ref: no) | 1.29 (1.16-1.42) | <0.001 |  | 1.31 (1.11-1.55) | <0.001 | 0.918 |
| Early morning awakening (ref: no) | 1.13 (1.01-1.26) | <0.001 |  | 1.33 (1.15-1.53) | <0.001 | 0.104 |
| Difficulty maintaining sleep (ref: no) | 1.22 (1.12-1.32) | <0.001 |  | 1.27 (1.15-1.41) | <0.001 | 0.631 |

Abbreviations: HR, hazard ratio; CI, confidence interval.

Adjusted for age, sex, ethnicity, education level, current smoking, alcohol drinking, BMI, regular exercise, hypertension, diabetes, stroke, cancer, chronic lung disease, heart disease, and depressive symptom.

**eTable 3. Association Between the Cumulative Number and Type of Insomnia Symptoms and IADL Disability Stratifying by Sex.**

| **Respondent Characteristics** | **Female** | |  | **Male** | | ***P* for interaction** |
| --- | --- | --- | --- | --- | --- | --- |
|  | **HR (95% CI)** | ***P* Value** |  | **HR (95% CI)** | ***P* Value** |  |
| Number of insomnia symptoms (ref: no symptoms) | | | | | | 0.077 |
| 1 | 1.14 (1.04-1.25) | 0.004 |  | 1.16 (1.04-1.29) | 0.007 |  |
| 2 | 1.14 (1.00-1.31) | 0.048 |  | 1.37 (1.15-1.63) | <0.001 |  |
| 3-4 | 1.10 (0.93-1.30) | 0.273 |  | 1.41 (1.11-1.80) | 0.005 |  |
| Individual insomnia symptoms | | | | | |  |
| Non-restorative sleep (ref: no) | 1.13 (1.00-1.27) | 0.044 |  | 1.21 (1.05-1.40) | 0.010 | 0.380 |
| Difficulty initiating sleep (ref: no) | 1.00 (0.89-1.12) | 0.995 |  | 1.22 (1.03-1.45) | 0.025 | 0.069 |
| Early morning awakening (ref: no) | 1.03 (0.91-1.16) | 0.663 |  | 1.32 (1.14-1.53) | <0.001 | 0.010 |
| Difficulty maintaining sleep (ref: no) | 1.16 (1.06-1.26) | 0.001 |  | 1.16 (1.04-1.29) | 0.009 | 0.687 |

Abbreviations: HR, hazard ratio; CI, confidence interval.

Adjusted for age, sex, ethnicity, education level, current smoking, alcohol drinking, BMI, regular exercise, hypertension, diabetes, stroke, cancer, chronic lung disease, heart disease, and depressive symptom.

**eTable 4. Association Between the Trajectories of Insomnia Symptoms and ADL Disability Stratifying by Sex.**

| **Trajectories of Insomnia Symptoms** | **Female** | |  | **Male** | | ***P* for interaction** |
| --- | --- | --- | --- | --- | --- | --- |
|  | **HR (95% CI)** | ***P* Value** |  | **HR (95% CI)** | ***P* Value** |  |
| Low | 1.00 (reference) | - |  | 1.00 (reference) | - | 0.244 |
| Decreasing | 1.16 (1.04-1.30) | 0.010 |  | 1.34 (1.16-1.55) | <0.001 |  |
| Increasing | 1.19 (0.99-1.43) | 0.067 |  | 1.28 (0.99-1.66) | 0.063 |  |
| High | 1.39 (1.18-1.64) | <0.001 |  | 1.22 (0.94-1.60) | 0.140 |  |

Abbreviations: HR, hazard ratio; CI, confidence interval.

Adjusted for age, sex, ethnicity, education level, current smoking, alcohol drinking, BMI, regular exercise, hypertension, diabetes, stroke, cancer, chronic lung disease, heart disease, and depressive symptom.

**eTable 5. Association Between the Trajectories of Insomnia Symptoms and IADL Disability** **Stratifying by Sex.**

| **Trajectories of Insomnia Symptoms** | **Female** | |  | **Male** | | ***P* for interaction** |
| --- | --- | --- | --- | --- | --- | --- |
|  | **HR (95% CI)** | ***P* Value** |  | **HR (95% CI)** | ***P* Value** |  |
| Low | 1.00 (reference) | - |  | 1.00 (reference) | - | 0.160 |
| Decreasing | 1.05 (0.93-1.18) | 0.433 |  | 1.30 (1.12-1.50) | <0.001 |  |
| Increasing | 1.07 (0.88-1.30) | 0.484 |  | 1.10 (0.83-1.45) | 0.514 |  |
| High | 1.02 (0.84-1.22) | 0.863 |  | 1.06 (0.80-1.41) | 0.691 |  |

Abbreviations: HR, hazard ratio; CI, confidence interval.

Adjusted for age, sex, ethnicity, education level, current smoking, alcohol drinking, BMI, regular exercise, hypertension, diabetes, stroke, cancer, chronic lung disease, heart disease, and depressive symptom.

**eTable 6. Association Between the Cumulative Number and Type of Insomnia Symptoms and ADL Disability after Excluding Participants Missing Covariate Data.**

| **Respondent Characteristics** | **Events/Total** | **Model 1^a^** | |  | **Model 2^b^** | |
| --- | --- | --- | --- | --- | --- | --- |
|  |  | **HR (95% CI)** | ***P* Value** |  | **HR (95% CI)** | ***P* Value** |
| Number of insomnia symptoms (ref: no symptoms) | | | | | | |
| 1 | 1 186/2 723 | 1.32 (1.23-1.41) | <0.001 |  | 1.23 (1.15-1.32) | <0.001 |
| 2 | 448/878 | 1.69 (1.53-1.87) | <0.001 |  | 1.46 (1.32-1.62) | <0.001 |
| 3-4 | 254/509 | 1.68 (1.48-1.91) | <0.001 |  | 1.43 (1.25-1.63) | <0.001 |
| Individual insomnia symptoms | | | | | | |
| Non-restorative sleep (ref: no) | 569/1 222 | 1.35 (1.24-1.48) | <0.001 |  | 1.31 (1.20-1.43) | <0.001 |
| Difficulty initiating sleep (ref: no) | 568/1 099 | 1.59 (1.46-1.74) | <0.001 |  | 1.32 (1.21-1.44) | <0.001 |
| Early morning awakening (ref: no) | 554/1 163 | 1.41 (1.29-1.55) | <0.001 |  | 1.23 (1.12-1.34) | <0.001 |
| Difficulty maintaining sleep (ref: no) | 1 230/2 654 | 1.37 (1.28-1.46) | <0.001 |  | 1.25 (1.17-1.34) | <0.001 |

Abbreviations: HR, hazard ratio; CI, confidence interval.

^a^ Unadjusted.

^b^ Adjusted for age, sex, ethnicity, education level, current smoking, alcohol drinking, BMI, regular exercise, hypertension, diabetes, stroke, cancer, chronic lung disease, heart disease, and depressive symptom.

**eTable 7. Association Between the Cumulative Number and Type of Insomnia Symptoms and IADL Disability after Excluding Participants Missing Covariate Data.**

| **Respondent Characteristics** | **Events/Total** | **Model 1^a^** | |  | **Model 2^b^** | |
| --- | --- | --- | --- | --- | --- | --- |
|  |  | **HR (95% CI)** | ***P* Value** |  | **HR (95% CI)** | ***P* Value** |
| Number of insomnia symptoms (ref: no symptoms) | | | | | | |
| 1 | 1 064/2 723 | 1.26 (1.18-1.36) | <0.001 |  | 1.16 (1.08-1.25) | <0.001 |
| 2 | 374/878 | 1.44 (1.29-1.61) | <0.001 |  | 1.22 (1.10-1.37) | <0.001 |
| 3-4 | 214/509 | 1.47 (1.27-1.68) | <0.001 |  | 1.20 (1.04-1.38) | 0.012 |
| Individual insomnia symptoms | | | | | | |
| Non-restorative sleep (ref: no) | 477/1 222 | 1.19 (1.08-1.31) | <0.001 |  | 1.16 (1.05-1.27) | 0.003 |
| Difficulty initiating sleep (ref: no) | 465/1 099 | 1.37 (1.24-1.51) | <0.001 |  | 1.08 (0.98-1.19) | 0.136 |
| Early morning awakening (ref: no) | 489/1 163 | 1.35 (1.23-1.48) | <0.001 |  | 1.15 (1.04-1.27) | 0.004 |
| Difficulty maintaining sleep (ref: no) | 1 077/2 654 | 1.27 (1.18-1.36) | <0.001 |  | 1.15 (1.08-1.24) | <0.001 |

Abbreviations: HR, hazard ratio; CI, confidence interval.

^a^ Unadjusted.

^b^ Adjusted for age, sex, ethnicity, education level, current smoking, alcohol drinking, BMI, regular exercise, hypertension, diabetes, stroke, cancer, chronic lung disease, heart disease, and depressive symptom.

**eTable 8. Association Between the Trajectories of Insomnia Symptoms and ADL Disability after Excluding Participants Missing Covariate Data.**

| **Trajectories of Insomnia Symptoms** | **Events/Total** | **Model 1^a^** | |  | **Model 2^b^** | |
| --- | --- | --- | --- | --- | --- | --- |
|  |  | **HR (95% CI)** | ***P* Value** |  | **HR (95% CI)** | ***P* Value** |
| Low | 1 289/4 741 | 1.00 (reference) | - |  | 1.00 (reference) | - |
| Decreasing | 748/2 077 | 1.38 (1.26-1.51) | <0.001 |  | 1.24 (1.13-1.36) | <0.001 |
| Increasing | 182/505 | 1.42 (1.22-1.66) | <0.001 |  | 1.25 (1.07-1.46) | 0.006 |
| High | 236/608 | 1.56 (1.35-1.79) | <0.001 |  | 1.37 (1.18-1.58) | <0.001 |

Abbreviations: HR, hazard ratio; CI, confidence interval.

^a^ Unadjusted.

^b^ Adjusted for age, sex, ethnicity, education level, current smoking, alcohol drinking, BMI, regular exercise, hypertension, diabetes, stroke, cancer, chronic lung disease, heart disease, and depressive symptom.

**eTable 9. Association Between the Trajectories of Insomnia Symptoms and IADL Disability after Excluding Participants Missing Covariate Data.**

| **Trajectories of Insomnia Symptoms** | **Events/Total** | **Model 1^a^** | |  | **Model 2^b^** | |
| --- | --- | --- | --- | --- | --- | --- |
|  |  | **HR (95% CI)** | ***P* Value** |  | **HR (95% CI)** | ***P* Value** |
| Low | 1 216/4 741 | 1.00 (reference) | - |  | 1.00 (reference) | - |
| Decreasing | 663/2 077 | 1.28 (1.17-1.41) | <0.001 |  | 1.17 (1.06-1.29) | 0.001 |
| Increasing | 160/505 | 1.27 (1.08-1.50) | 0.004 |  | 1.07 (0.90-1.26) | 0.443 |
| High | 193/608 | 1.27 (1.09-1.48) | 0.002 |  | 1.11 (0.94-1.30) | 0.216 |

Abbreviations: HR, hazard ratio; CI, confidence interval.

^a^ Unadjusted.

^b^ Adjusted for age, sex, ethnicity, education level, current smoking, alcohol drinking, BMI, regular exercise, hypertension, diabetes, stroke, cancer, chronic lung disease, heart disease, and depressive symptom.

**eTable 10. Association Between** **the Cumulative Number and Type of Insomnia Symptoms and ADL Disability after Excluding Participants Who Developed ADL Disability During the First Two Years of Follow-up.**

| **Respondent Characteristics** | **Events/Total** | **Model 1^a^** | |  | **Model 2^b^** | |
| --- | --- | --- | --- | --- | --- | --- |
|  |  | **HR (95% CI)** | ***P* Value** |  | **HR (95% CI)** | ***P* Value** |
| Number of insomnia symptoms (ref: no symptoms) | | | | | | |
| 1 | 1 003/2 651 | 1.27 (1.18-1.37) | <0.001 |  | 1.19 (1.10-1.28) | <0.001 |
| 2 | 373/834 | 1.65 (1.48-1.84) | <0.001 |  | 1.44 (1.29-1.61) | <0.001 |
| 3-4 | 200/479 | 1.52 (1.32-1.75) | <0.001 |  | 1.34 (1.16-1.55) | <0.001 |
| Individual insomnia symptoms | | | | | | |
| Non-restorative sleep (ref: no) | 488/1 207 | 1.33 (1.21-1.46) | <0.001 |  | 1.31 (1.19-1.45) | <0.001 |
| Difficulty initiating sleep (ref: no) | 472/1 050 | 1.56 (1.42-1.71) | <0.001 |  | 1.32 (1.20-1.46) | <0.001 |
| Early morning awakening (ref: no) | 456/1 119 | 1.36 (1.23-1.50) | <0.001 |  | 1.19 (1.08-1.32) | <0.001 |
| Difficulty maintaining sleep (ref: no) | 996/2 506 | 1.29 (1.20-1.38) | <0.001 |  | 1.18 (1.10-1.27) | <0.001 |

Abbreviations: HR, hazard ratio; CI, confidence interval.

^a^ Unadjusted.

^b^ Adjusted for age, sex, ethnicity, education level, current smoking, alcohol drinking, BMI, regular exercise, hypertension, diabetes, stroke, cancer, chronic lung disease, heart disease, and depressive symptom.

**eTable 11. Association Between the Cumulative Number and Type of Insomnia Symptoms and IADL Disability after Excluding Participants Who Developed IADL Disability During the First Two Years of Follow-up.**

| **Respondent Characteristics** | **Events/Total** | **Model 1^a^** | |  | **Model 2^b^** | |
| --- | --- | --- | --- | --- | --- | --- |
|  |  | **HR (95% CI)** | ***P* Value** |  | **HR (95% CI)** | ***P* Value** |
| Number of insomnia symptoms (ref: no symptoms) | | | | | | |
| 1 | 934/2 703 | 1.23 (1.14-1.33) | <0.001 |  | 1.16 (1.08-1.26) | <0.001 |
| 2 | 316/850 | 1.37 (1.22-1.54) | <0.001 |  | 1.27 (1.12-1.42) | <0.001 |
| 3-4 | 168/491 | 1.26 (1.08-1.48) | 0.003 |  | 1.19 (1.01-1.39) | 0.003 |
| Individual insomnia symptoms | | | | | | |
| Non-restorative sleep (ref: no) | 402/1 221 | 1.10 (1.00-1.22) | 0.062 |  | 1.16 (1.05-1.29) | 0.004 |
| Difficulty initiating sleep (ref: no) | 387/1 072 | 1.28 (1.16-1.43) | <0.001 |  | 1.11 (1.00-1.24) | 0.046 |
| Early morning awakening (ref: no) | 401/1 129 | 1.24 (1.12-1.37) | <0.001 |  | 1.14 (1.03-1.26) | 0.015 |
| Difficulty maintaining sleep (ref: no) | 918/2 579 | 1.23 (1.14-1.32) | <0.001 |  | 1.17 (1.08-1.26) | <0.001 |

Abbreviations: HR, hazard ratio; CI, confidence interval.

^a^ Unadjusted.

^b^ Adjusted for age, sex, ethnicity, education level, current smoking, alcohol drinking, BMI, regular exercise, hypertension, diabetes, stroke, cancer, chronic lung disease, heart disease, and depressive symptom.

**eTable 12. Association Between the Trajectories of Insomnia Symptoms and ADL Disability after Excluding Participants Who Developed ADL Disability During the First Two Years of Follow-up.**

| **Trajectories of Insomnia Symptoms** | **Events/Total** | **Model 1^a^** | |  | **Model 2^b^** | |
| --- | --- | --- | --- | --- | --- | --- |
|  |  | **HR (95% CI)** | ***P* Value** |  | **HR (95% CI)** | ***P* Value** |
| Low | 1 168/5 051 | 1.00 (reference) | - |  | 1.00 (reference) | - |
| Decreasing | 635/2 104 | 1.32 (1.20-1.46) | <0.001 |  | 1.21 (1.10-1.34) | 0.005 |
| Increasing | 166/529 | 1.46 (1.24-1.72) | <0.001 |  | 1.27 (1.08-1.50) | <0.001 |
| High | 191/597 | 1.47 (1.26-1.71) | <0.001 |  | 1.33 (1.13-1.56) | <0.001 |

Abbreviations: HR, hazard ratio; CI, confidence interval.

^a^ Unadjusted.

^b^ Adjusted for age, sex, ethnicity, education level, current smoking, alcohol drinking, BMI, regular exercise, hypertension, diabetes, stroke, cancer, chronic lung disease, heart disease, and depressive symptom.

**eTable 13. Association Between the Trajectories of Insomnia Symptoms and IADL Disability after Excluding Participants Who Developed IADL Disability During the First Two Years of Follow-up.**

| **Trajectories of Insomnia Symptoms** | **Events/Total** | **Model 1^a^** | |  | **Model 2^b^** | |
| --- | --- | --- | --- | --- | --- | --- |
|  |  | **HR (95% CI)** | ***P* Value** |  | **HR (95% CI)** | ***P* Value** |
| Low | 1 203/5 051 | 1.00 (reference) | - |  | 1.00 (reference) | - |
| Decreasing | 616/2 104 | 1.26 (1.14-1.38) | <0.001 |  | 1.17 (1.06-1.29) | 0.002 |
| Increasing | 167/529 | 1.30 (1.10-1.54) | 0.002 |  | 1.08 (0.91-1.28) | 0.358 |
| High | 158/597 | 1.17 (1.00-1.38) | 0.057 |  | 1.03 (0.87-1.22) | 0.701 |

Abbreviations: HR, hazard ratio; CI, confidence interval.

^a^ Unadjusted.

^b^ Adjusted for age, sex, ethnicity, education level, current smoking, alcohol drinking, BMI, regular exercise, hypertension, diabetes, stroke, cancer, chronic lung disease, heart disease, and depressive symptom.

**eTable 14. Association Between the Cumulative Number and Type of Insomnia Symptoms and each** **ADL disability.**

| **Respondent Characteristics** | **HR (95% CI)** | | | | | |
| --- | --- | --- | --- | --- | --- | --- |
|  | **Difficulty in dressing** | **Difficulty in bathing** | **Difficulty in eating** | **Difficulty in using the toilet** | **Difficulty in getting in/out of bed** | **Difficulty in walking across a room** |
| Number of insomnia symptoms (ref: no symptoms) | | | | | | |
| 1 | 1.19 (1.11-1.28) | 1.20 (1.11-1.29) | 1.02 (0.92-1.12) | 1.20 (1.10-1.30) | 1.19 (1.09-1.29) | 1.14 (1.06-1.24) |
| 2 | 1.42 (1.28-1.57) | 1.26 (1.13-1.40) | 1.15 (1.00-1.32) | 1.38 (1.23-1.54) | 1.43 (1.27-1.61) | 1.31 (1.17-1.46) |
| 3-4 | 1.39 (1.23-1.57) | 1.18 (1.04-1.34) | 1.09 (0.93-1.28) | 1.29 (1.13-1.47) | 1.54 (1.35-1.76) | 1.25 (1.10-1.42) |
| Individual insomnia symptoms | | | | | | |
| Non-restorative sleep (ref: no) | 1.20 (1.10-1.31) | 1.11 (1.01-1.21) | 1.09 (0.97-1.22) | 1.19 (1.08-1.31) | 1.24 (1.13-1.37) | 1.15 (1.05-1.26) |
| Difficulty initiating sleep (ref: no) | 1.27 (1.16-1.39) | 1.25 (1.14-1.37) | 1.09 (0.97-1.22) | 1.20 (1.09-1.32) | 1.38 (1.25-1.52) | 1.29 (1.17-1.41) |
| Early morning awakening (ref: no) | 1.26 (1.16-1.38) | 1.08 (0.98-1.19) | 1.02 (0.91-1.15) | 1.12 (1.02-1.24) | 1.33 (1.20-1.46) | 1.08 (0.98-1.19) |
| Difficulty maintaining sleep (ref: no) | 1.22 (1.14-1.31) | 1.13 (1.05-1.22) | 1.09 (0.99-1.19) | 1.23 (1.14-1.33) | 1.24 (1.14-1.34) | 1.15 (1.07-1.24) |

Abbreviations: HR, hazard ratio; CI, confidence interval.

Adjusted for age, sex, ethnicity, education level, current smoking, alcohol drinking, BMI, regular exercise, hypertension, diabetes, stroke, cancer, chronic lung disease, heart disease, and depressive symptom.

**eTable 15. Association Between the Cumulative Number and Type of Insomnia Symptoms and each IADL disability.**

| **Respondent Characteristics** | **HR (95% CI)** | | | | |
| --- | --- | --- | --- | --- | --- |
|  | **Difficulty in using the phone** | **Difficulty in managing money** | **Difficulty in taking medications** | **Difficulty in shopping for groceries** | **Difficulty in preparing hot meals** |
| Number of insomnia symptoms (ref: no symptoms) | | | | | |
| 1 | 1.08 (0.99-1.18) | 1.05 (0.96-1.13) | 1.05 (0.96-1.16) | 1.15 (1.07-1.25) | 1.11 (1.01-1.21) |
| 2 | 1.05 (0.92-1.20) | 1.13 (1.00-1.27) | 1.26 (1.11-1.43) | 1.26 (1.12-1.41) | 1.20 (1.06-1.36) |
| 3-4 | 1.05 (0.91-1.23) | 1.08 (0.95-1.24) | 1.08 (0.93-1.26) | 1.27 (1.11-1.45) | 1.19 (1.03-1.38) |
| Individual insomnia symptoms | | | | | |
| Non-restorative sleep (ref: no) | 0.98 (0.88-1.09) | 0.98 (0.89-1.08) | 1.03 (0.92-1.15) | 1.12 (1.02-1.23) | 1.16 (1.04-1.28) |
| Difficulty initiating sleep (ref: no) | 1.18 (1.06-1.31) | 1.13 (1.03-1.25) | 1.19 (1.07-1.33) | 1.18 (1.07-1.30) | 1.16 (1.04-1.28) |
| Early morning awakening (ref: no) | 1.07 (0.96-1.19) | 1.12 (1.02-1.24) | 1.12 (1.00-1.25) | 1.18 (1.07-1.30) | 1.12 (1.01-1.25) |
| Difficulty maintaining sleep (ref: no) | 0.99 (0.90-1.08) | 1.05 (0.97-1.13) | 1.04 (0.95-1.13) | 1.16 (1.07-1.25) | 1.09 (1.00-1.19) |

Abbreviations: HR, hazard ratio; CI, confidence interval.

Adjusted for age, sex, ethnicity, education level, current smoking, alcohol drinking, BMI, regular exercise, hypertension, diabetes, stroke, cancer, chronic lung disease, heart disease, and depressive symptom.

**eTable 16. Association Between the Trajectories of Insomnia Symptoms and each ADL disability.**

| **Trajectories of Insomnia Symptoms** | **HR (95% CI)** | | | | | |
| --- | --- | --- | --- | --- | --- | --- |
|  | **Difficulty in dressing** | **Difficulty in bathing** | **Difficulty in eating** | **Difficulty in using the toilet** | **Difficulty in getting in/out of bed** | **Difficulty in walking across a room** |
| Low | 1.00 (reference) | 1.00 (reference) | 1.00 (reference) | 1.00 (reference) | 1.00 (reference) | 1.00 (reference) |
| Decreasing | 1.14 (1.04-1.25) | 1.12 (1.02-1.23) | 0.99 (0.88-1.12) | 1.10 (0.99-1.22) | 1.14 (1.02-1.27) | 1.10 (0.99-1.21) |
| Increasing | 1.01 (0.86-1.18) | 1.25 (1.08-1.44) | 1.08 (0.90-1.29) | 1.27 (1.08-1.49) | 1.29 (1.09-1.51) | 1.18 (1.00-1.38) |
| High | 1.26 (1.10-1.45) | 1.17 (1.03-1.34) | 0.95 (0.81-1.13) | 1.35 (1.17-1.56) | 1.33 (1.15-1.55) | 1.29 (1.13-1.49) |

Abbreviations: HR, hazard ratio; CI, confidence interval.

Adjusted for age, sex, ethnicity, education level, current smoking, alcohol drinking, BMI, regular exercise, hypertension, diabetes, stroke, cancer, chronic lung disease, heart disease, and depressive symptom.

**eTable 17. Association Between the Trajectories of Insomnia Symptoms and each IADL disability.**

| **Trajectories of Insomnia Symptoms** | **HR (95% CI)** | | | | |
| --- | --- | --- | --- | --- | --- |
|  | **Difficulty in using the phone** | **Difficulty in managing money** | **Difficulty in taking medications** | **Difficulty in shopping for groceries** | **Difficulty in preparing hot meals** |
| Low | 1.00 (reference) | 1.00 (reference) | 1.00 (reference) | 1.00 (reference) | 1.00 (reference) |
| Decreasing | 0.98 (0.88-1.10) | 1.02 (0.92-1.13) | 0.90 (0.80-1.01) | 1.18 (1.05-1.31) | 1.09 (0.97-1.23) |
| Increasing | 0.90 (0.75-1.08) | 1.08 (0.92-1.27) | 0.87 (0.72-1.05) | 1.03 (0.86-1.23) | 1.00 (0.82-1.22) |
| High | 1.01 (0.86-1.18) | 1.06 (0.91-1.23) | 0.94 (0.80-1.11) | 1.09 (0.93-1.28) | 1.13 (0.96-1.34) |

Abbreviations: HR, hazard ratio; CI, confidence interval.

Adjusted for age, sex, ethnicity, education level, current smoking, alcohol drinking, BMI, regular exercise, hypertension, diabetes, stroke, cancer, chronic lung disease, heart disease, and depressive symptom.
